# Can a lifestyle intervention be offered through NHS breast cancer screening? Challenges and opportunities identified in a qualitative study of women attending screening

**DOI:** 10.1186/s12889-016-3445-7

**Published:** 2016-08-11

**Authors:** Ellie Conway, Sally Wyke, Jacqui Sugden, Nanette Mutrie, Annie S. Anderson, on behalf of the ActWELL team

**Affiliations:** 1 Institute of Health and Wellbeing, College of Social Science, University of Glasgow, 27 Bute Gardens, Glasgow, G12 8RS UK; 2 Centre for Public Health Nutrition Research, Ninewells Hospital & Medical School, University of Dundee, Level 7, Dundee, DD2 4BF UK; 3 Physical Activity for Health Research Centre, The University of Edinburgh, St Leonard’s Land, Holyrood Road, Edinburgh, EH8 8AQ UK; 4 Centre for Research into Cancer Prevention and Screening, Ninewells Medical School, University of Dundee, Level 7, Mailbox 7, Dundee, DD1 9SY UK

**Keywords:** Lifestyle, Breast cancer screening, Alcohol, Body-weight, Physical activity

## Abstract

**Background:**

Around one third of breast cancers in post-menopausal women could be prevented by decreasing body fatness and alcohol intake and increasing physical activity. This study aimed to explore views and attitudes on lifestyle intervention approaches in order to inform the proposed content of a lifestyle intervention programme amongst women attending breast cancer screening.

**Methods:**

Women attending breast cancer screening clinics in Dundee and Glasgow, were invited to participate in focus group discussions (FGD) by clinic staff. The groups were convened out with the clinic setting and moderated by an experienced researcher who attained brief details on socio-demographic background and audio-recorded the discussions. Data analysis was guided by the framework approach. The main topics of enquiry were: Understanding of risk of breast cancer and its prevention, views on engaging with a lifestyle intervention programme offered through breast cancer screening and programme design and content.

**Results:**

Thirty one women attended 5 focus groups. Participant ages ranged from 51 to 78 years and 38 % lived in the two most deprived quintiles of residential areas. Women were generally positive about being offered a programme at breast cancer screening but sceptical about lifestyle associated risk, citing genetics, bad luck and knowing women with breast cancer who led healthy lifestyles as reasons to query the importance of lifestyle. Engagement via clinic staff and delivery of the programme by lifestyle coaches out with the screening setting was viewed favourably. The importance of body weight, physical activity and alcohol consumption with disease was widely known although most were surprised at the association with breast cancer. They were particularly surprised about the role of alcohol and resistant to thinking about themselves having a problem. They expressed frustration that lifestyle guidance was often conflicting and divergent over time. The concept of focussing on small lifestyle changes, which were personalised, supported socially and appropriate to age and ability were welcomed.

**Conclusions:**

Offering access to a lifestyle programme through breast screening appears acceptable. Explaining the relevance of the target behaviours for breast cancer health, endorsing and utilising consistent messages and identifying personalised, mutually agreed, behaviour change goals provides a framework for programme development.

## Background

Breast cancer accounted for 15 % of all cancer diagnoses in Scotland in 2012 [1]. It is estimated that 38 % of post-menopausal breast cancer could be prevented by increased physical activity and reductions in alcohol and body fatness [2]. Whilst it is not possible to conduct long term randomised controlled trials of primary prevention to demonstrate that changes in these behaviours will decrease incidence of the disease, a systematic review identified that intentional weight loss is associated with a decreased incidence of cancer, particularly female obesity-related cancers such as breast cancer [3]. In addition, moderating weight gain in adult life through caloric adjustment (including calories from alcoholic drinks as well as food and other drinks) and physical activity is likely to be of benefit for reduction in cancers related to these behaviours (notably colon cancer) as well as other non-communicable diseases [4–6]

Lifetime weight gain is a strong predictor of breast cancer notably in women who have not taken hormone replacement therapy [7]. Ahn et al. [8] reported that at any BMI, weight gain in adult life is associated with greater risk of breast cancer and a gain of 2–10 kg after the age of 50 is associated with a 30 % increase in breast cancer risk. In the Women’s Health Initiative, Neuhouser et al. [9] reported that in post-menopausal women with a BMI < 25 kg/m^2^ at baseline who gained > 5 kg of body weight during the follow up period (median 13 years) had a 36 % increase risk of developing breast cancer. It is notable that whilst high weight gain in midlife has been associated with an increased risk of breast cancer diagnosis before or at age 50 in women, [10] a recent meta-analysis has reported that adult weight gain was unrelated to cancers of the breast in premenopausal women (and in postmenopausal HRT users [11].

These data provide a good rationale to support lifestyle change (notably behaviours that impact on weight management) to reduce breast cancer risk. However, but there is little evidence that known associations between breast cancer risk and lifestyle behaviours have been either widely communicated or had a major influence on behavioural choices. For example, a 2010 survey conducted by Cancer Research UK identified that although cancer is the UK’s number one health fear, “more than a third think getting the disease is down to fate and there is nothing they can do to avoid it” [12]. These beliefs are likely to have major implications for determining subsequent behaviour to reduce risk [13]. There is little evidence of breast cancer risk reduction campaigns within the NHS or third sector; whilst many cancer charities raise awareness about screening and treatments, there are few programmes actively involved in lifestyle prevention specifically focusing on weight loss in relation to breast cancer.

Around 75 % of Scottish women aged 50 to 70 years accept invitations to attend the Scottish NHS breast screening programme (NHSSBSP) with over 160,000 women seen annually [14]. In addition, women aged over 70 are able to attend through self-referral. We have worked in conjunction with the NHSSBSP to develop, refine and conduct a feasibility trial of a lifestyle intervention programme, ActWELL [15], which aims to help women make small, sustainable changes to improve physical activity, alcohol consumption and diet in order to reduce their future risk of breast cancer.

The ActWELL programme was based on best evidence of which behavioural change techniques are most effective in increasing physical activity and improving diet including setting individual weight management goals (weight loss or avoidance of weight gain) [16]. The specific techniques used were setting long-term weight management goals (weight loss or avoidance of weight gain), behavioural goals for everyday eating and physical activity, problem solving, action planning and self-monitoring of steps. A full description of the intervention format is available elsewhere [17].

The programme development also informed by qualitative research exploring the views of health professionals, managers, charity workers about the possible implementation of the ActWELL intervention in routine practice and in everyday life and observations within NHS screening services. In addition, we used by used data from previous lifestyle interventions in colorectal cancer settings [17–19] which were shown to achieve **successful** changes in lifestyle, were acceptable to participants and feasible to deliver. These approaches included implementation intention, self-monitoring and telephone contact. The intervention aimed to help women increase physical activity, modify their diet, lower their alcohol intake and set individual weight management goals (weight loss or avoidance of weight gain) [19].

In this paper we present the results of formative analysis of qualitative data with women who had previously attended routine breast screening clinic in order to share some key factors which shaped our intervention that we believe would be useful for other researchers planning work in this field.

## Methods

### Participants and recruitment

Women attending routine breast cancer screening clinics in Dundee and Glasgow (the target group for the proposed intervention) were invited to participate in a focus group discussion (FGD) by NHSBC clinic receptionists. Radiographers were asked to endorse study participation after the mammographic procedures and collected contact details of women willing to participate, which were forwarded to the research team. The research team telephoned women to check their availability, and, if they were still willing to take part, arranged a suitable time for them to attend a FGD in a university setting.

### Data collection and analysis

The topic guide focussed on three key concepts relevant to intervention design:

1. Risk of breast cancer and its prevention. We wanted to know what women already knew about cancer, and the values and attitudes they held towards the disease and its causes and perceptions about lifestyle interventions
2. Views on engaging with a lifestyle intervention programme offered through breast cancer screening
3. Views on intervention programme design (delivery, content).

Written information was provided on estimated breast risk from lifestyle and a prototype programme design used for discussion. The estimated risk figures used were population based as published by the World Cancer Research Fund and available on the web for the general public [2]. These were not individualised according to family history. Excess body fat is clearly identified as a risk factor, as is alcohol and physical activity and each were described in written materials. The prototype described an intervention of one face to face session (with a trained ActWELL coach) of one hour duration plus follow up telephone calls offering support and feedback on behaviour change goals. The proposed topics were those relevant for breast cancer prevention namely weight management, alcohol and physical activity. The intervention was planned to be personalised and to use behaviour change techniques (described above) to support and facilitate change. To assist discussion on body weight, coloured BMI charts (http://www.vertex42.com/ExcelTemplates/bmi-chart.html) for self-assessment of BMI category (link) was also used to discuss aspects of weight management.

An experienced researcher facilitated FGDs which were audio recorded with written informed consent and transcribed. Individuals’ contribution to the discussion was not identified with pseudonyms or numbers as part of the anonymization process. All participants were also invited to complete a brief questionnaire (age, postcode, height and weight) prior to commencing the FGD. Post code was used to assess Scottish Index of Multiple Deprivation (SIMD) – a categorical system of identifying social position based on area of residence which takes account of housing, crime, access to services, education, health, income and employment [20].

The second author read a sample of transcripts and agreed a final coding frame which was then applied to the data. Data analysis was guided by the Framework approach [21], a form of structured, thematic analysis that allows for the pre-identification of initial themes whilst allowing unanticipated, emergent themes to be identified.

Ethical approval was provided by the East of Scotland Research Ethics Service, REC reference no. 12/ES/0087. All participants provided written, informed consent.

## Results

Table 1 illustrates that 31 women attended 5 focus groups, three of which took place in Dundee and two in Glasgow. Two participants failed to provide personal details. Participant age ranged from 51 to 78 years, and they had a mean estimated BMI of 26.2 kg/m^2^ (range 20 to 41). Overall, 38 % lived in the two most deprived SIMD quintiles.

**Table 1:** Background socio-demographic information of participants

	FG 1 *n =* 8	FG 2 *n =* 2	FG 3 *n =* 7	FG 4 *n =* 7	FG 5 *n =* 5
Date of FG	11.03.13	14.03.13	15.03.13	18.03.13	19.03.13
Venue	Dundee	Glasgow	Dundee	Glasgow	Dundee
Age (years) Mean (Range)	65.0 (51–78)	63.5 (59–68)	64.0 (52–73)	56.1 (52–61)	57.8 (51–66)
BMI (kg/m^2^) Mean (Range)	29.3 (22–41)	23.5(22–25)	24.6 (20–33)	28.3 (25–33)	24.2 (21–30)
Scottish Index of Multiple deprivation (SIMD)					
^a^1	2	1	0	3	1
2	1	0	0	3	0
3	1	0	1	1	1
4	3	0	3	0	2
5	1	1	3	0	1

^a^Most deprived neighbourhoods

### Risk of breast cancer and its prevention

Within all focus groups there was a general awareness of factors which increased risk of breast cancer and a general acceptance that lifestyle behaviours play a role in the aetiology of the disease. However, women also highlighted that heredity was the most significant risk in any form of cancer highlighted by such phrases as *“if you are prone to it you are going to get it…” (FG2)*. A common thread of fatalism was also apparent, interwined with the genetic explanation. Comments in FG1, such as “*if it’s for you, it won’t go by (FG1) and* “*Sometimes it just seems the luck of the draw…” (FG1)* illustrate views suggesting that fate or luck played a central role in causation.

### Views on engaging with a lifestyle intervention programme offered through breast cancer screening

All the women had been provided with written information about ActWell by receptionists in the clinics, and this was then followed up by radiographers in private consultation while conducting the mammogram. Women appreciated this approach and the relaxed, non-pressured way that the topic was raised by both reception staff and radiographers:

*“the radiographer was just the back-up – ‘were you handed the leaflet’, and I said yes” (FG3)*

In most FGD women felt the breast cancer screening clinic was a very good place to be invited to a lifestyle programme because women there were already thinking of breast cancer risk:

“*that's why we’re all here, because of breast cancer…so it does relate straight back” (FG1)*

*“it’s like an emphasis, here’s another positive thing you can do” (FG5)*

Although in other FGD there was discussion about the possibility of raising anxiety and also that the screening was a difficult enough experience as it was:

*“Some people are very, very anxious when they are going to have the breast screening” (FG2)*

*“It is painful so I don’t think it’s the right setting. I was glad to get out of there” (FG5)*

One focus group in particular highlighted a potential to increase anxiety for some women in the gap between attending for screening and receiving results, by planting seeds in their minds that their own lifestyle might have contributed to a diagnosis. This anxiety, compounded by a lack of time on the part of radiographers and appropriate locations to have private conversations between patients and clinic staff, meant that it was suggested that there are better moments to provide the actual intervention than at routine screening appointments such as a follow up visit.

Participants in the focus groups were however, generally positive about the design of the ActWell intervention, in particular the aims to make small changes to their lifestyles with the objective of maintaining weight or achieving small to moderate weight loss. There was a general consensus that small aims were both realistic and achievable for individual women.

Discussion tended to focus mainly on the support element of the programme, and how women would prefer to be supported while they were taking part.

“*For myself, a group, more than a one to one, with two or three folk has always motivated me a bit more because you get ideas from other people” (FG1)*

However, equally there was enthusiasm for the one to one support of an ActWell support worker:

“*having contact with someone makes you think, well, maybe I will do it” (FG3)*

*“it’s personalised to you, you don’t have to face a group” (FG5)*

Having face to face contact with the lifestyle coach was seen as especially beneficial, and women drew on anecdotes about how advice received in person – for example at ante-natal classes – had stuck with them over time, rather than if it had been transmitted through written material. Moreover, meeting face to face meant there was a personal relationship between participant and coach which would be of benefit:

“*somebody sat down and talked to me about it…so it kind of went into my head…I think it was because somebody else was taking the time to spend and explain it to me” (FG5)*

“*if you do it one to one you can explore the hindrances, like what stops you doing it” (FG5)*

### Programme design and content

It was notable that women described encouragement to engage in lifestyle change as ‘preachy’ or nagging. It was clear that women felt ill at ease about health messages which they had repeatedly been exposed to in the past: *“that’s the same three things [diet, exercise and alcohol] that they are always hitting you with” (FG4).* There was also a sense of exasperation that the content of messages was perceived to be inconsistent, and therefore unreliable:

*“they say one thing one month and then six months later they say, oh don’t do that because it’s not good for you” (FG3)*

*“they tell you at one point this is really not good for you…then maybe four or five years down the line they will say, no, it’s really ok, you can have that” (FG4)*

Not only did this result in frustration among the participants, but also a loss of confidence in those disseminating these messages conveyed in comments such as *“I think if the professionals can’t get it right, why do we bother?” (FG1).*

Women also reported a sense of overload of messages (many of which were perceived as negative) *“I think in general people are fed up to the back teeth being told that this is not healthy and that’s not healthy and you mustn’t eat this and you can’t eat it, this is negative health messages and I think that is not good” (FG5).* There was a sense that *“folk just start to switch off…” (FG1)* from health messages and *“[People will] do what they want to do” (FG3)* or ignore it all completely *reporting* that *I’ll just go on as I’m doing possibly” (FG2)*

### Specific lifestyle topics

Whilst it was not surprising to participants that body weight, physical activity and alcohol consumption all play a role in cancer risk the direct link between them and breast cancer was surprising. Several participants expressed doubt about the validity of the statistics, and both acceptance and equivocation were reinforced by anecdote and personal experience:

*“I’m…thinking to myself, my sister had breast cancer about 2011, she’s the very opposite to me, she’s slim…she’s very active, she doesn’t drink, she’s not overweight, she got cancer. I’m the opposite and I haven’t” (FG1)*

### Alcohol

Most of the participants who took part in the focus groups reported drinking low to moderate levels of alcohol. Most agreed that alcohol consumption was a sensitive subject in general with one group noting that “*it’s trickier [to talk about] than smoking” (FG4)”.* Moreover, it was felt that many people tend to downplay or underestimate their own alcohol intake illustrated by a wide range of comments including *“A lot of people hide it” (FG5)* and *“they have some perception that they are going to be judged, even if they are not a heavy drinker” (FG4)*

It was generally thought that alcohol was a predominant and inescapable feature in modern life, and that *“Everybody drinks” (FG4)*, whether in a social context or at home. For example “*It’s become a social thing, hasn’t it, women get together and say bring a bottle of wine, then they have one glass, they have another glass and they forget just how much they are taking” (FG1).* Because alcohol plays such a deeply embedded role in social relationships it was suggested that particular attention should be paid to balancing messages, to avoid being perceived as preaching or controlling.

Keeping track of personal alcohol intake was seen as challenging for individuals, not least because of difficulties estimating volume of alcohol consumed and confusion over alcohol units. The middle-aged women in the FGDs expressed the view that whilst *they* did not generally have a problem with alcohol it was a big problem for *young people* and in FG3 they broadened the topic out to the Scottish Government’s plans to introduce minimum unit pricing on alcohol:

*Participant 1 in exchange: And because they [the Scottish Government] have this agenda to reduce, well I think its young people who are drinking too much and then causing violence.*

*Participant 2 in exchange: They’re never going to, you cannot stop people drinking, I don’t care what the Scottish government think they’re going to.*

So the topic of breast cancer risk and alcohol was transformed into a discussion of the problem that alcohol causes more broadly and was resolutely among young people, although it was noted in FG1 that “*I think the ones who are having the big glasses of wine and thinking its ok are our age group”.*

### Body weight

Many participants were surprised to find there was a direct link between breast cancer risk and body weight. The majority of those taking part had struggled with their weight, either throughout life or following menopause, and most were either overweight or obese. There was no awareness that menopause itself was associated with weight gain, although there were some references to an expectation of becoming heavier as one ages.

*“I got through the menopause really with no flushing, I never went on HRT, and I was really fine; but the weight…” (FG3)*

*“My weight crept up during the menopause but I didn’t know that it was the menopause I was going through” (FG5)*

The discussions illustrate that many women struggle with post-menopausal weight gain. Many women reported a history of dieting, either informally or through attending commercial slimming clubs. However, such efforts were deemed only temporarily successful, with weight rising once contact with the group or adherence to stricter plans loosened. Motivations to lose weight were described as changing over time. One woman described how, as a younger woman, she associated keeping her weight under control with physical attractiveness. Now, however, she had begun to lose weight gradually over time in order to improve her health

“*I lost two stone. I mean I feel so much younger and more active and I didn’t have any back pain, [or pain] in my knees and my ankles” (FG5)*

Her commitment to lose weight very gradually was indicative of an important factor for many of the participants in light of the ease of weight gain and difficulty in losing it they had experienced, which was to have achievable targets and realistic expectations of sustainable changes:

### Physical activity

Women did not distinguish physical activity for weight loss with physical activity for general reasons. They did describe participating in a range of activities, ranging from gardening to running and climbing and daily routines which incorporated some element of physical activity such as twice-daily dog-walking and taking grandchildren to school. While the participants all agreed that being physically active had unquestionable health benefits, several mentioned the social aspect of their physical activity, both in formal and informal settings:

*“I did it with a friend and each week we could share, have we succeeded, have we not succeeded, what we have found difficult, what we didn’t. (FG4)*

*“You get to meet different women, different sizes” (FG5)*

Participants said that it was important that activity was encouraged within the context of women’s particular lives - “*in an everyday kind of situation” (FG2);* that enjoyment was central - *“I think it’s the fun element that attracts people” (FG5);* and that motivation should ultimately focus on the individual - *“[doing it] for themselves” (FG2).* Yet some participants found their ability to exercise was restricted most commonly as a result of physical impairment or ailment. Comments referring to comorbidities such as *“I’m really restricted because of the spasms in my back and the tiredness and the pain” (FG4)* and *“I’ve knackered my knees and I’ve got a touch of arthritis” (FG5)* highlight some of the challenges of being physically active amongst older women. Even in the absence of specific medical problems, participants felt that age and its effect on lifestyle impacted on both their attitude towards exercise generally, and the type of activity they would be uncomfortable doing such as “*hectic or vigorous activity”.*

These views affected attitudes towards formal settings such as health clubs and the gym, which were seen as often financially unaffordable, but also unappealing:

“*Who’s got the money to go and join a gym?” (FG3)*

*“I think even if you want to go to the gym there’s not a lot of option for anybody our age, because it’s all spin fit, whatever…too much geared to young people” (FG4)*

Reported lack of time, personal preferences and sensitivities might nudge people towards less formal forms of exercise, specifically walking. However, potential barriers to regular walking included the climate and the social surrounding.

*“I find in the winter it’s not just exercise, the winter I don’t want to go out the door, so my treat…if I’m not working…is a full day in the house. But in the summertime it’s different…it’s brighter in the morning so you are up and you’re doing more” (FG4)*

## Discussion

The aim of focus group discussions is to seek the opinions of a range of people at the same time, and to examine points at which individual views converge and conflict. Overall, there was a high level of consensus, both within and between focus groups in each of the areas explored. While this might be attributed the nature of these groups discussions, participants were all strangers to one another and did not appear reticent in voicing contrasting positions (particularly on more sensitive topics) to contribute to the overall discussion.

It is clear that any intervention focussed on reducing breast cancer risk factors needs to be take account of beliefs around genetics, fate and personal experience. Whilst these beliefs have been described in women who are known to be at increased risk [22] or have had a breast cancer diagnosis [23] data on the views of women attending routine breast cancer screening have not previously been reported. The concept of a lifestyle intervention in breast cancer screening was generally well received by discussants who saw it as attractive with achievable and realistic aims. It was notable that all the participants were themselves recruited through breast screening clinics, and given the enthusiasm that participants displayed, both for taking part in the focus groups and for the intervention itself, it could be argued that offering a separate follow up appointment (rather than an intervention at the time of screening) offered a more favourable opportunity for intervention.

The provision of information in the waiting area prior to the mammogram enabled women to begin to reflect on the study, while the radiographer acted as an additional prompt was said to work well. This approach might address any concerns that radiographers would feel uncomfortable when raising the opportunity for a lifestyle intervention during their time-restricted consultations with individual women. Furthermore, participants in the focus groups described this approach as relaxed and informative thus it is plausible that a whole team approach to recruitment and engagement would be acceptable by screening attendees

The most significant concern about the nature of support offered was whether this would be best provided one to one, or in a group setting. It was recognised that are issues with setting up groups such as dovetailing busy schedules for multiple participants. The concept of ActWell coaches and receiving personal support on a one to one basis was viewed positively. These findings underlines the desire for advice and support to be given within what is perceived to be a reciprocal and respectful relationship. It is likely that both (group and individual) approaches are plausible for recruitment and engagement but ways to facilitate social support (which is associated with improved behaviour change adherence [24]) within one to one programmes should be explored.

There was evidence of confusion and frustration about public health lifestyle guidance which was seen as conflicting and changed over time. These findings suggest the need to maximise the opportunity to emphasise agreement from a multi-agency stakeholder group (e.g. NHS professionals, cancer charities and academic researchers as well as Scottish government (funders)) for the intervention in order to underline both the importance and consistency of messaging or perhaps explaining why lifestyle advice does change so rapidly.

Whilst women liked the idea of being offered support for lifestyle change, their suspicion of public health interventions in general, suggest there may be some resistance to elements of the programme. This was manifested as a questioning of the science underpinning the association between bodyweight, alcohol consumption and cancer risk. Social psychological research would suggest that they may have responded defensively to the personal risk implied [25]. Alcohol discussions were difficult for participants, and some may have underestimated their intake, not deliberately but because it was challenging to identify what constitutes a unit of alcohol. In common with participants in other studies of understanding of risk of illness and of health promotion messages [26, 27] women also seemed to distance themselves from the risk of breast cancer posed by drinking alcohol by emphasising that it is ‘others’ who are most at risk, and in this case, young people through alcohol consumption. This finding implies that there is still a need for robust public health communications before the messages based on the epidemiology of breast cancer risk are incorporated as every day, ‘common sense’ aspects of people’s understanding of cancer risk [28]. The recent media coverage of the proposed revision to UK alcohol guidelines highlights some of the challenges to women in achieving that understanding [29].

It is useful to consider how these findings might shape the intervention dialogue. With respect to alcohol it seems appropriate to offer practical guidance such as a measuring guide for units of alcohol which would provide women with a tool for monitoring intake and raise awareness of their own alcohol consumption. In addition, the provision of clear, information that illustrates the scientific evidence about the link between lifestyle factors and breast cancer risk has the potential to increase credibility of advice both in the context of alcohol as a carcinogen and indirectly through the role of alcoholic drinks in increasing caloric intake and potential contributor to weight gain.

Participants recognised the importance of physical activity, especially in connection with weight management but also described a range of personal, social and economic limitations to becoming more active. These findings highlight the importance of acknowledging what women already do, what they might like to do to increase physical activity and providing help to attain and sustain meaningful and practical goals. Women argued that changes to lifestyles as they aged were influenced by social roles and acknowledged it is easy to put on weight as their roles changed. They were, however, enthusiastic about the role of a lifestyle coach, who they thought would make the process of making changes easier (in part) due to personalised support.

This study has several limitations. While these women were drawn from a cross section of the general population, it should be noted that they were recruited via breast cancer screening clinics, and were arguably already actively concerned about their health and wellbeing. This means that, in interpreting the results of the study we need to be aware that respondents were likely to be those most keen on the idea of supporting breast cancer risk reduction. It is possible that respondents who did not offer to take part were likely to be much more sceptical. Nevertheless, we are confident that participants were not just telling us what we wanted to hear given the breadth of challenges, issues and questioning of the links between weight, alcohol consumption and breast cancer risk.

## Conclusion

Offering access to a lifestyle programme through breast screening appears acceptable. Explaining the relevance of the target behaviours for breast cancer health, endorsing and utilising consistent messages and identifying mutually agreed, behaviour change goals provides a basic framework for programme development**.** These findings add to the existing evidence base on effective behaviour change techniques and highlight the need for personalised gender and age specific approaches to realising the potential of breast screening as an opportunity for a “teachable moment” [30] for breast cancer risk reduction.

## Abbreviations

FGD, Focus Group Discussion, NHSSBSP, NHS Scotland breast screening programme, NPT, Normalization Process Theory
